# High-throughput nitrogen-vacancy center imaging for nanodiamond photophysical characterization and pH nanosensing†

**DOI:** 10.1039/d0nr05931e

**Published:** 2020-10-26

**Authors:** Maabur Sow, Horst Steuer, Sanmi Adekanye, Laia Ginés, Soumen Mandal, Barak Gilboa, Oliver A. Williams, Jason M. Smith, Achillefs N. Kapanidis

**Affiliations:** Biological Physics Research Group, Department of Physics, University of Oxford Oxford OX1 3PU UK kapanidis@physics.ox.ac.uk; Department of Materials, University of Oxford Parks Road Oxford OX1 3PH UK; School of Physics and Astronomy, Cardiff University Cardiff CF24 3AA UK

## Abstract

The fluorescent nitrogen-vacancy (NV) defect in diamond has remarkable photophysical properties, including high photostability which allows stable fluorescence emission for hours; as a result, there has been much interest in using nanodiamonds (NDs) for applications in quantum optics and biological imaging. Such applications have been limited by the heterogeneity of NDs and our limited understanding of NV photophysics in NDs, which is partially due to the lack of sensitive and high-throughput methods for photophysical analysis of NDs. Here, we report a systematic analysis of NDs using two-color wide-field epifluorescence imaging coupled to high-throughput single-particle detection of single NVs in NDs with sizes down to 5–10 nm. By using fluorescence intensity ratios, we observe directly the charge conversion of single NV center (NV^−^ or NV^0^) and measure the lifetimes of different NV charge states in NDs. We also show that we can use changes in pH to control the main NV charge states in a direct and reversible fashion, a discovery that paves the way for performing pH nanosensing with a non-photobleachable probe.

## Abstract 2

A method to observe individual fluorescent crystal defects in nanodiamonds is reported and opens new nanosensing avenues (*e.g.* pH nanosensing).

## Graphical abstract

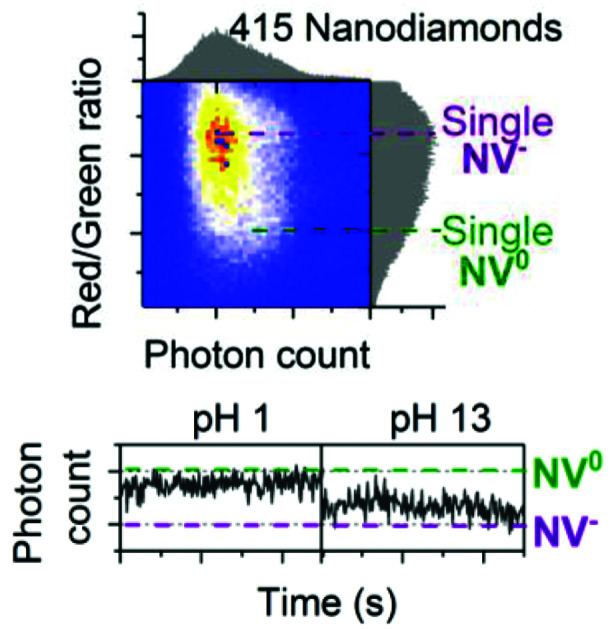

## Introduction

**Fig. 1:**
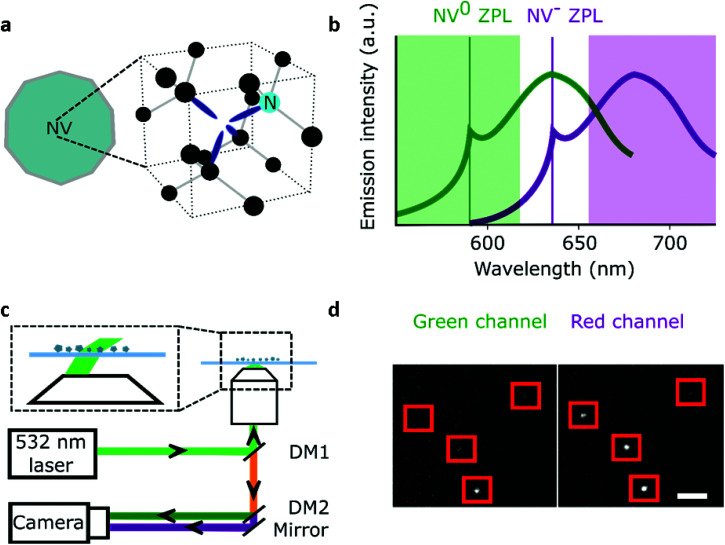
Wide-field imaging of NDs. a, Schematic representation of a ND containing a NV center including the atomic structure of the defect. b, Emission spectrum of NV^−^ measured in the 44 nm NDs and a schematic of the blue-shifted emission of the neutral state NV^0^ (ZPL: zero-phonon line). The green highlighted rectangle (550–620 nm) corresponds to the spectral region collected by the green channel, while the red rectangle (655–750 nm) is the spectral region collected for the red channel. c, Simplified representation of the wide-field microscope with NDs immobilised on a glass microscope slide. The zoomed-in region shows variable-angle illumination; DM1: dichroic with reflective bands at 532 and 638 nm; DM2: 650 nm long-pass dichroic. d, Section of the field of view of surface immobilised fluorescent NDs. 532 nm excitation at 7.8 kW cm^−2^, 100 ms exposure; the red squares represent NDs localised by our software (Gapviewer). The photon count for each ND per camera exposure is calculated from a region of interest centred on the fluorescent spot; scale bar = 8 μm.

Nanodiamonds (NDs) as single-photon sources and bio-imaging probes have attracted significant interest in the past two decades.^1–3^ A major reason for this attention is that, for NDs with a diameter of 35 nm or more, the fluorescent nitrogen-vacancy (NV) centers in the ND emit bright photoluminescence without blinking or photobleaching.^4^ The NV crystal defect consists of a substitutional nitrogen atom adjacent to a carbon vacancy (Fig. 1a) that can be incorporated inside NDs (5–200 nm) at different concentrations;^5^ moreover, NDs show little toxicity and are easy to functionalize.^6^ These desirable properties have been exploited in *in vitro* single-molecule experiments and long (>10 min) intracellular tracking;^7–11^ such tracking also enabled nanosensing inside living cells.^12,13^

A facile way for nanosensing is to detect changes in the charge of the NV center as charge transitions (NV^−^ and NV^0^) are triggered by chemical events or variations of electrical potential.^14,15^ A more sensitive sensing approach exploits the spin-state-dependent fluorescence of the NV center, which can be manipulated at room temperature;^16–18^ this property was harnessed to measure temperature changes inside living neurons.^19^*In vitro* experiments also demonstrated that NDs can be used to detect down to a few atomic spin labels (*i.e.*, gadolinium atoms), whereas a single nitroxide label inside a protein was sensed using NV center in a bulk diamond.^20,21^

Despite their promise, ND applications in bioimaging have been limited by the low brightness of sub 20 nm NDs, since a single NV center is 10-times less bright than a typical organic fluorophore used in single-molecule fluorescence detection. Moreover, it is still very difficult to manufacture small NDs suitable for high-sensitivity nanosensing (*i.e.*, magnetic field from individual molecules or atoms), since such NDs must contain few impurities (*e.g.*, nitrogen or ^13^C) and little crystal strain. As a result, there are numerous efforts to manufacture small, bright, and high-purity NDs that differ by size, nitrogen content and surface chemistry; these ND samples display different NV center emission spectra and intensity levels due to interactions with the surface, or due to a different number of NV centers per particle.^22,23^ For these reasons, the photophysical characterization of NDs is crucial to ensure successful applications in bioimaging, especially using single-molecule microscopy. Further, since NDs are increasingly implemented in quantum technologies, the characterization of NV centres in NDs is an important endeavor, as most of our knowledge on the photophysics of the NV centre originated from studies performed in bulk diamond.^24–27^

An important question in ND characterization is the proportion of NDs containing single NV centers, a property paramount for the optimization of ND manufacture and for ND applications as single-photon sources.^2,28^ The conventional method used for confirming the presence of the single NV centers is the measurement of the coherence of NV fluorescence emission and the calculation of the probability of photons being emitted at the same time. However, such photon-correlation experiments require complex instrumentation and have limited throughput, since each measurement needs to be performed individually on single NDs.^29,30^ An alternative method to identify single NV center relies on measuring the photon count corresponding to a single NV center; this, however, is also complicated by the orientation of the two NV center's orthogonal dipoles.^25^ As a result, there is a need for a high-throughput method reporting on the fraction of single emitters in NDs.

Characterization methods are also essential for new ND bio-sensing assays. For instance, Raabova *et al.* showed that the charge state of a NV center in polymer-coated NDs could be used to detect pH changes;^31^ this was achieved by measuring changes in the ND's emission spectra to differentiate the two photoactive charge states of the NV center (NV^−^ and NV^0^; NV^+^ is non-photoactive), since NV^0^ has its emission 60 nm blue-shifted compared to NV^−^ (see Fig. 1b).^32^ The same team also established that the charge state of NV centers in NDs is affected by specific chemical changes on the ND surface caused by pH or temperature variations. Other reports using a similar approach showed that it is possible to detect different chemical changes on the ND surface (*e.g.*, modification of functional groups or adsorption of DNA *etc*.);^14,33,34^ however, direct charge manipulation of single NV centers by pH in ND was not performed because the NDs used were too large (∼49 nm).^34^ Indeed, 10–20 nm NDs would be optimal for such experiments, since they contain “shallower” NV centers – however, working with small NDs requires sensitive equipment to detect single NVs.^14^

Here, we report a sensitive and high-throughput wide-field ratiometric imaging approach that allows us to measure reliably single NV charge states and compare the proportion of single emitters in different ND samples (5–200 nm in size). This ratiometric approach is based on a similar approach that has been used extensively to perform single-molecule Förster Resonance Energy transfer (FRET) measurements on biomolecules;^35–38^ here, we have adapted the ratiometric approach to study NV photophysics in a robust and high-throughput manner. Our approach provides hundreds of ND fluorescence time-traces in seconds, which allows measurements of the proportion of NDs containing single NV centers; further, the ratiometric nature of our measurements allows detection of spectral shifts that unravel changes in the NV center charge state. We use our method to observe directly dynamic charge-state transitions in multiple NDs, and to demonstrate that the charge state in 10 nm NDs can be directly and reversibly manipulated by pH, making NDs promising probes for pH sensing.

## Materials and methods

### Nanodiamond samples

Doped nanodiamonds of 10, 40, 44 and 200 nm in diameter were commercial high-pressure-high temperature (HPHT) samples enriched in nitrogen followed by particle irradiation to generate vacancies (providers: 10 and 40 nm: Adámas nano; 44 nm: FND biotech; 200 nm: Columbus Nanoworks). Following the high-temperature annealing of NDs, the vacancies recombine with the nitrogen to form NV centers. When not provided by the manufacturer, the fraction of NDs containing NV was coarsely estimated by dividing the density of fluorescent NDs by the density of NDs deposited on the glass surface. The values obtained from 2 to <0.1% are within the range of values reported in the literature (0.03 to 70%) depending on the size and manufacturing process.^5,39^ The 44 nm NDs were manufactured to contain a maximum proportion of one NV center per ND. The NV center's emission spectra were measured in all our samples (*e.g.*, Fig. S12†). The presence of single quantum emitters in the 44 nm sample was confirmed by photon-correlation experiments (Fig. S3†). Our 50 nm NDs were undoped HPHT particles (from Microdiamant) and the 5 nm detonation particles obtained from PlasmaChem GmbH; ND monodisperse suspensions were prepared from the raw powders (for the 5- and 50 nm samples) by our teams. All the samples were acid-cleaned, sonicated and their size distribution was confirmed by single-particle tracking and/or dynamic light scattering. The 10 nm doped ND have a nominal size of 10 nm; however, our analysis revealed a size distribution centered at 20 nm; this discrepancy may be due to the irregular shapes of small NDs (such as flakes).^40^

### Microscope and imaging

The particles were spin-coated at a very low density (down to 1 fluorescent ND for 40 μm^2^) and imaged using a single-molecule desktop wide-field microscope (Nanoimager S, Oxford Nanoimaging) with a 1.4 NA oil-immersion objective that provided a ∼80μm diameter excitation spot. The emitted light was split into a green and red imaging channels with a long pass filter at 650 nm for the red channel (see Fig. 1c). The dichroics mentioned in Fig. 1 are produced by Semrock (reference: DM1: FF545/650-Di01-25x36; DM2: FF640-FDi01-25x36). The 1 W 532 nm CW laser allowed us to detect single NV centers with 10–1000 ms time resolution when used at full intensity (7.8 kW cm^−2^). The camera is a sCMOS type from Hamamatsu (reference C13440-20CU). The exposure time of 100 ms was selected for most experiments, as it provided the best SNR for a minimal acquisition time (25 s). Illumination for the TIRF objective was set at ∼50° to remove out-of-focus background. All the samples were exposed to maximum excitation intensity for 30–60 s to photobleach other emitting species (*e.g.*, surface defects). The photon count distribution from 80 to 300 particles per sample was collected by imaging different fields of view. A custom-built confocal set-up was used to perform spectral measurement and photon correlation experiments as we previously reported.^41^

For pH sensing, the 10 nm NDs were immersed in different buffer solutions using silicone gaskets to form wells. A 0.01 M HCl solution was used for pH 1.2; a 0.1 M NaOH solution for pH 12.8–12.9; a 1× phosphate-buffered saline solution (PBS) for pH 7; a commercial buffer solution (Hanna instruments) was used for pH 4 and 10. Washing was performed using deionized and filtered water (220 nm pores filter), and the acquisition was done 1–3 min following the solution addition. The accuracy of our pH measurement was estimated by averaging the residuals, with
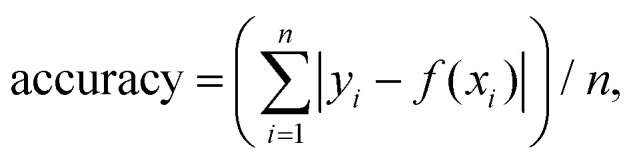
where *n* is the number of data points, *y* is the pH value measured using a pH meter, and *f*(*x*) is the pH value estimated using the linear fit of the measured R/G ratio (*i.e.*, the variable *x*) to the pH values; for better clarity, *x* and *y* axes in Fig. 5b are inverted compared to the analysis described above.

### Time-trace analysis and HMM

The raw image was processed by home-built software (GapViewer) that detects diffraction-limited spots by their intensity. It performs local background subtraction for each frame using the intensity around the Gaussian profile of the NDs emission. The photon count is calculated by adding both channels and the R/G ratio was computed as R/G ratio = Spot Intensity_red_/(Spot Intensity_red_ + Spot Intensity_green_). Based on the NV center emission spectra of the two charge states previously reported^24^ and the wavelength dependence of the quantum efficiency from the microscope's sCMOS camera (Fig. S2†), we expect a R/G ratio difference of 0.3 between NV^−^ and NV^0^, with NV^0^ being brighter than NV^−^. The dynamic traces were manually selected based on their R/G ratio (within 0.6 and 0.9) and their intensity (0–1500 photons per s for the green channel and 1000–3200 photons per s for the red channel). Some traces (<10%) were excluded from the analysis if their R/G ratio transitions were showing more than 3 states because it could indicate the presence of a second, less bright NV center. Following HMM processing (ebFRET, 10 restarts, 0.001 precision and prior strength set with a 0.2 center (priors set only to extract dwell-times)), the durations of the dwell-times for each state were extracted and fitted with a single or double exponential decay.

## Results

### High-throughput analysis of different ND samples

To study the number of NV/ND and their charge state of NV in a parallel, high-throughput manner, we used a wide-field approach combined with two-channel imaging as a way to obtain simultaneously intensity and spectral information on the NDs. Spectral insight is gained by performing a ratiometric measurement on each NDs, by utilizing our two imaging channels: our “red channel” (which detects fluorescence in the red to far-red part of the spectrum) and our “green channel” (which detects fluorescence in the green to red part of the spectrum). Indeed, NV^−^ mostly emits fluorescence in the red channel, while NV^0^ emits to roughly the same degree in the green and red channels (Fig. 1b). We thus devised a simple ratio (see below) using the intensities in the two imaging channels to monitor the spectrum of single NVs, and in turn, to monitor their charge state. To validate our approach, we analyzed samples with an expected large difference in the distribution between NV^0^ and NV^−^.

The NDs we used have different sizes (5–200 nm diameter), manufacturing processes and NV center content (see Methods). The 5 and 10 nm diameter NDs are usually too small to contain more than 2 NVs per ND, and include only a small fraction of NDs (up to ∼1%) that carry an NV center.^39^ Since they are undoped, the 50 nm diameter NDs are expected to have up to ∼10% of fluorescent NDs containing only 1–2 NV centers per particle.^22^ The doped 40- and 44 nm diameter NDs contain up to 4 NV/ND and show a larger fraction of bright NDs (up to 70% for the 40 nm, see Methods). Finally, the 200 nm doped NDs are all expected to be emitting fluorescence and can have up to 100 NV/ND because of their larger size.

The large field of view (50 × 80 μm) of the microscope allowed us to use a low ND density (down to one fluorescent ND for 40 μm^2^, Fig. 1d), ensuring that we observe single NDs (Fig. S1†). We collected 96 to 589 time-traces per sample and used all data points to build the photon count distribution of the sum of red and green channels. To detect changes in the emission spectrum, we compared the relative intensities of the red and green channels using the R/G ratio: (R/G ratio = Spot Intensity_red_/(Spot Intensity_red_ + Spot Intensity_green_)).

Based on the reported spectra (for both charge states), we expected that the R/G ratio for NV^−^ will be ∼0.3 higher than the R/G ratio for NV^0^.^24^

**Fig. 2:**
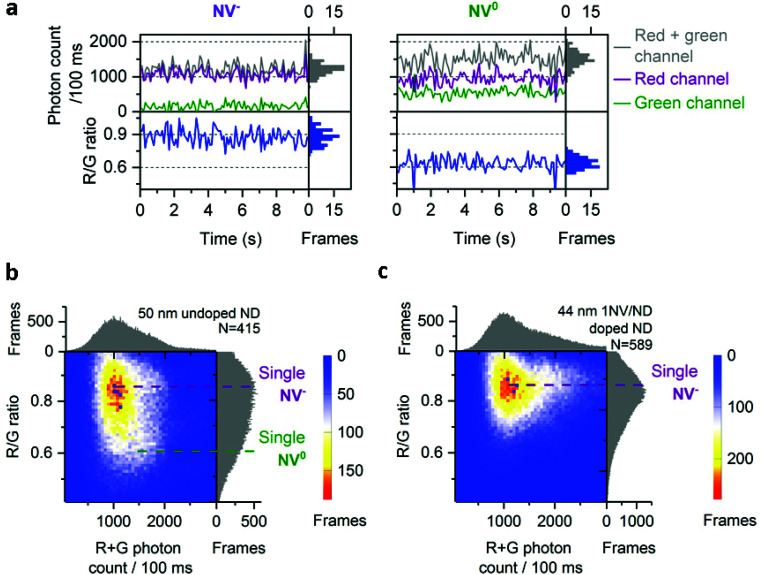
Charge states of NV center in doped and undoped NDs. a, Typical time-traces from 50 nm undoped NDs corresponding to single NV^−^ and NV^0^. b, 2D histogram of photon count and R/G ratio in 50 nm undoped NDs; purple and green dotted lines indicate respectively single NV^−^ or NV^0^ fluorescence. c, 2D histogram of photon count and R/G ratio in 44 nm 1 NV/ND doped NDs; the purple line indicates the fluorescence from single NV^−^. For (b) and (c), *N* is the number of fluorescent NDs observed. For all the figures, “Frames” refers to the frequency of detection of a ND particle in single frames (see Fig. 1 for details). The immobilized NDs were imaged in air with 532 nm excitation at 7.8 kW cm^−2^ and 100 ms exposures for 25 s.

For the 50 nm undoped NDs, most time-traces showed a total photon count of 1000/100 ms and a R/G ratio of 0.9 (Fig. 2a, left; Fig. 2b). Other traces were brighter (1500 photons per 100 ms) with a lower R/G ratio (0.6), as more light is detected in the green channel (Fig. 2a, right). These two populations with distinct R/G ratio can also be seen as a very broad R/G ratio distribution on a 2D histogram (Fig. 2b). We attributed these two R/G ratio states to the different charge states of the NV center (0.6 for NV^0^ and 0.9 for NV^−^) based on their respective emission spectra and our detection efficiency for the red and green channels (Fig. S2†). The photon counts we obtained are 20-fold higher than previously reported for a single NV center using wide-field imaging.^42^

**Fig. 3:**
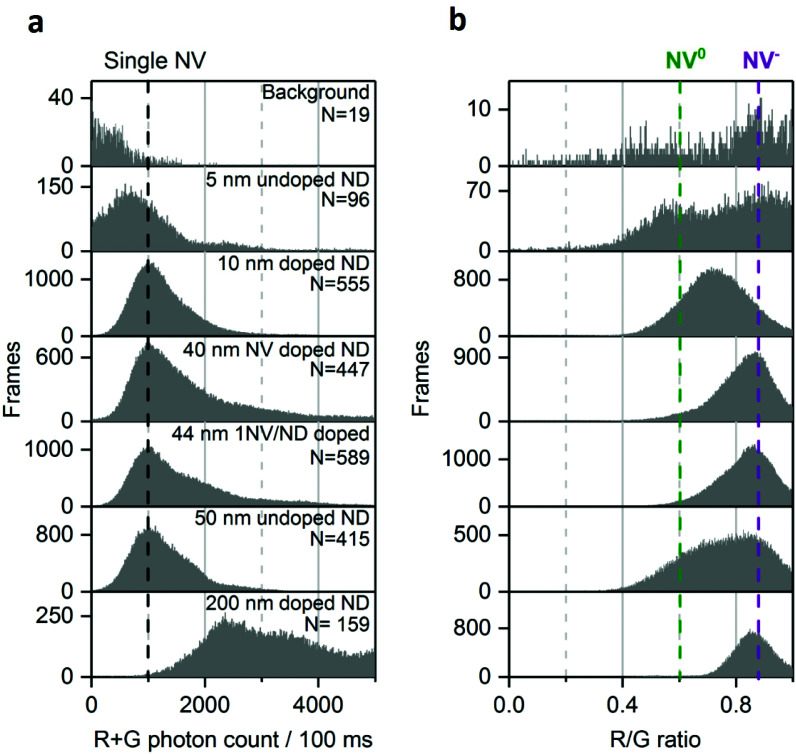
Distribution of photon count (a) and R/G ratio (b) in different NDs samples. Black dotted line marks the photon count collected from single NV center. The background signal is the fluorescence originating from impurities in the microscope slide. The purple and green dotted lines indicate NV^−^ and NV^0^ fluorescence, respectively. Using multiple-Gaussian fitting, the fraction of single NV center (NV^−^ or NV^0^) per ND is >70% for the 50 nm undoped and 10 nm doped NDs, <50% for the 44 and 40 nm-doped NDs and 0% for the 200 nm doped NDs. Similar analysis for the 5 nm undoped NDs is not possible because of their low brightness and low statistics. *N* is the number of fluorescent NDs observed and “Frames” refers to the frequency of detection of a ND particle in single frames (see Fig. 1 for details). The immobilized NDs were imaged in air with 532 nm excitation at 7.8 kW cm^−2^ and 100 ms exposures for 25 s.

The 2D histogram for 44 nm NDs shows the same main population as the 50 nm undoped NDs (Fig. 2c); this similarity allows us to assign this intensity to a single NV^−^ emission, since this sample was manufactured to contain 1NV/ND; we further verified the presence of single NV^−^ per ND in the 44 nm NDs using photon correlation experiments (Fig. S3†). Notably, no clear NV^0^ signal at a R/G ratio of 0.6 is observed in these 44 nm NDs, which confirms that the population with a 0.6 R/G ratio in Fig. 2b is NV^0^. The proportion of NV^−^ in doped NDs is expected to be high (*e.g.* >65% in 40 nm NDs according to the supplier) as ND doping involves high concentration of nitrogen (up to 200 ppm) to improve the probability of NV center formation; high nitrogen content is known to stabilize NV's negative charge state as nitrogen acts as an electron donor for NV centers.^25^ Similar distributions of photon count and R/G ratio were measured in 40 nm doped NDs (Fig. 3a and b, row 5), an expected result based on the comparable size and manufacturing process for the 40 and 44 nm doped NDs.

We then examined sub-20 nm NDs, which are challenging samples as they contain more unstable NV charge states.^39,43^ In such small particles, the fluorescent defect is closer to charge traps at the surface that can act as electron acceptors to NV^−^.^44^ We observed that the photon count of the 10 nm doped NDs mainly originates from single NV centers, as expected given their small size (Fig. 3a, row 3); the distribution of the R/G ratio is centered around 0.7 (Fig. 3b, row 3), suggesting that the charge conversion occurs within 100 ms. We attribute this charge state instability to the NV center's proximity to the surface.

The 5 nm NDs show low brightness, a small bright fraction (<1%; see Methods) and, unlike all the other samples, most photobleach within seconds. Nonetheless, we can confirm the rare presence of stable and bright NDs (>2000 photons per 100 ms; Fig. 3a, row 2), as previously reported.^39,43^ Given the scarcity of such emitters (<0.1% of the total 5 nm NDs) one cannot exclude the possibility that we detected fluorescence from a small subpopulation of NDs having a larger size than 5 nm (*e.g.*, 10–30 nm, as reported by Vlasov *et al.*).^43^

The 200 nm doped NDs exhibit a very broad photon count distribution (Fig. 3, bottom; showing only NDs with photon count <5000 photons per 100 ms, ∼20% of the 200 nm NDs). The first maximum of the photon count distribution is >2000 photons per 100 ms, indicating that the sample contains no single NVs per ND, consistent with the probability of having NDs containing one NV center being very low in such large doped NDs. Finally, no NV^0^ fluorescence is present in the R/G distribution (Fig. 3b, bottom), very likely due to their size and high nitrogen concentration.

### Dynamic behavior of NDs

**Fig. 4:**
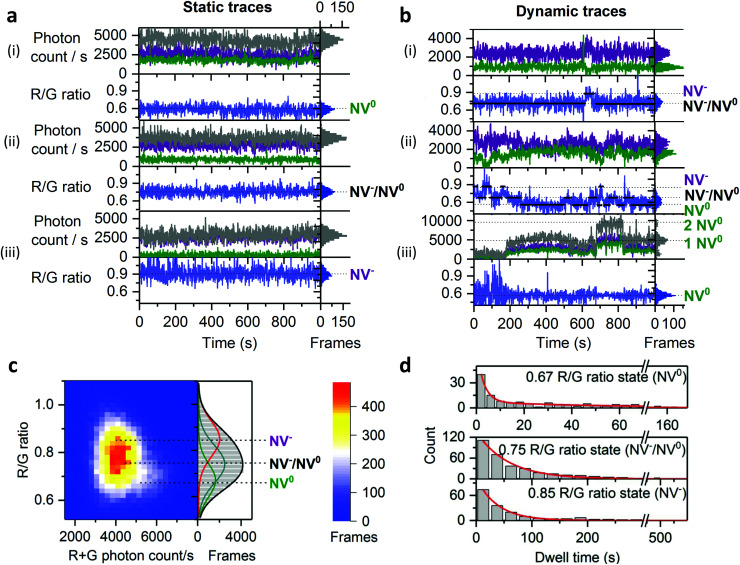
States and dynamics of NV center in NDs. a, Typical static time-traces corresponding to the two different charge states of single NV centers ((i): NV^0^ R/G ratio = 0.6–4000 R + G photons per s; (iii): NV^−^: R/G ratio = 0.9–2000 R + G photons per s) and a time-average of the two charge states ((ii): NV^−^/NV^0^: R/G ratio = 0.7–0.8 – 2000–4000 R + G photons per s). Red and green photon counts are shown in purple and green, respectively; the sum of red and green photon counts is in gray. b, Typical dynamic traces; the first trace (i) shows two-state transitions as demonstrated by the spectral inversion; the second trace (ii) is more dynamic and shows three-state transitions; the last trace (iii) shows two-level blinking c, 2D histogram of 32 dynamic traces with their R/G ratio distribution on the right side of the figure; the R/G distribution is fitted with 3 Gaussian profiles centered at the R/G ratio values calculated by HMM modelling, which fits well the distribution and the 2D histogram especially the brighter NV^0^ population. Gaussian centers: NV^0^ R/G ratio = 0.67; time-averaged NV^−^/NV^0^: R/G ratio = 0.75; NV^−^: R/G ratio = 0.85. d, Dwell-time analysis of the 3 states by HMM. The distribution of dwell-times is fitted with a double exponential decay function for the NV^0^ state (top histogram, fitting function shown in red). The lifetimes are *τ*_1_ = 3 s ± 0.3 s *A*_*τ*_1__ = 90%; *τ*_2_ = 53 s ± 7.7 s *A*_*τ*_2__ = 10% for NV_0_. The distribution of dwell-times is fitted with a single-exponential decay function for the NV^−^/NV^0^ and NV^−^ states (middle and bottom histograms). The lifetimes are: *τ* = 56 s ± 1.7 s for NV^−^/NV^0^; *τ* = 38 s ± 1.5 s for NV^−^. “Frames” refers to the frequency of detection of a ND particle in single frames. The immobilized NDs were imaged in air using 532 nm excitation at 3.4 kW cm^−2^ and 1 s exposures for 20 min to 60 min.

To capture dynamic transitions in NDs occurring in the timescale of seconds, we studied 40 nm NDs exhibiting NV charge-state transitions. For the population with single NVs, we measured steady fluorescence in 60% of them (Fig. 4a) as opposed to the remaining 40%, in which clear dynamic behavior was observed. The vast majority of dynamic traces are due to NV centers with unstable charge states (Fig. 4b(i) and (ii)); further, a very small fraction (<1% of the total traces) showed on/off blinking (Fig. 4b(iii)). According to previous studies, fluctuations in ND fluorescence are due to the proximity (<10 nm) between the NV center and the ND's surface;^29,45^ electron transfer from the NV center to surface charge traps allows a NV defect to switch from NV^−^ to NV^0^ and then to the non-photoactive NV^+^ charge state, causing blinking.

To study the dynamic traces, we first defined the charge states in terms of photon count and R/G ratio using stable time-traces (Fig. S4†). We assigned emitters having a photon count of 4000 s^−1^ and a R/G ratio of 0.6 to single NV^0^ (16% of static traces, Fig. 4a, top). We also assigned the traces showing a photon count between 2000 and 4000 photons per s and a R/G ratio of 0.7 to 0.8 to time-averaged values of the NV^−^ and NV^0^ states (38% of static traces, Fig. 4a, middle); such averaging is consistent with charge conversion in bulk diamonds that may occur within the μs timescale.^46^ Finally, we assigned the NDs emitting 2500 photons per s with a R/G ratio of 0.9 to single NV^−^ (48% of static traces; Fig. 4a, bottom).

We then collected long dynamic traces (each lasting 30–60 min), which provided enough statistics to compare state transitions within NDs (*N* = 32, providing >600 dwells). The dynamic traces showed either two-state or three-state transitions (Fig. 4b(i) and (ii)) with the transition frequency varying among NDs (Fig. S5†). To investigate if these dynamic NDs share similar states and dwell-times, we performed Hidden Markov modelling (HMM) analysis, which showed that a three-state model was sufficient to fit our dynamic traces (Fig. S8†);^47^ the three states identified (NV^−^ at 0.85, NV^0^ at 0.67 and a time-averaged state at 0.75, Fig. 4c) correspond well to those seen in static traces. The slight difference (±0.07) between the values from the static and dynamic traces is likely due to small errors in the state allocation by HMM.

We used the dwell-times from the HMM analysis to calculate the lifetimes of each NV charge state. Dwell-time distributions from the NV^−^ and NV^−^/NV^0^ states were fitted with a single-exponential decay function (Fig. 4b, middle and bottom histograms), while the dwell-time distribution from the NV^0^ state had to be fitted with a double-exponential decay function (Fig. 4b top histogram) as the single-exponential decay was clearly missing a long-dwell component. Most of the NV^0^ dwells (∼90%) are less than 10 s, leading to a lifetimes of *τ*_1_ ∼ 3 s, which is substantially shorter than the two other states (*τ* ∼ 57 s for NV^−^/NV^0^ and *τ* ∼ 38 s for NV, Fig. 4d); this result is likely to reflect the use of high 532 nm excitation, a wavelength known to pump NV^0^ back to NV^−^, thus making NV^0^ lifetime shorter in our imaging conditions.^24,48^

### Charge state manipulation using pH

**Fig. 5:**
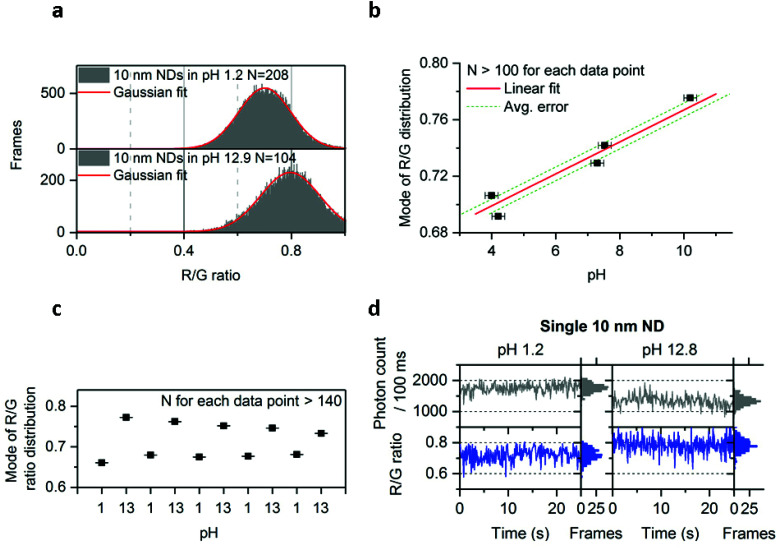
Effect of pH on 10 nm NDs. a, Increase of the R/G ratio distribution in the 10 nm doped NDs at high pH. b, Relation of the mode of the R/G ratio distribution with pH following Gaussian fitting of the distribution. Data points from pH 4 to 10 are fitted with a linear function, with the average error corresponding to an average of the absolute value of residuals (*i.e.*, the difference between data points and the predicted value from the fit; see Methods for more details). Duplicates were pulled to make the data points shown in this figure. *X* error bars are obtained from the pH meter's accuracy; *Y* error bars (smaller than the black squares icon) are the accuracy from the Gaussian fit c, Reversibility of the effect following multiple washing on the same field of view containing more than 140 NDs (washing was performed using deionized and filtered water; the same acidic/basic solutions as in figure a were used). The convergence of the modes after the first repeat may be due to a change in the ND's surface chemistry. d, Observation of the charge state conversion on the same ND particle in acidic and basic solutions (same solutions used as in figure a). R + G photon count is shown here. Approximately 75% of the single NV centers observed showed this increase of the R/G ratio in basic solution. The error bars (Fig. 5b and c) indicate the standard error from the Gaussian fitting. *N* is the number of fluorescent NDs observed and “Frames” refers to the frequency of detection of a ND particle in single frames (see Fig. 1 for details). See Methods for details about the solutions used. The immobilized NDs were imaged with 532 nm excitation at 7.8 kW cm^−2^ and 100 ms exposures for 25 s.

Since we could detect different charge states of the NV center, we investigated our ability to modify the ND's emission by subjecting them to treatments that may affect the chemical and physical properties of the ND surface. We first subjected immobilized 10 nm NDs to treatments using separate solutions containing DNA, proteins, and a reducing agent (DTT); none of these solutions showed detectable effects on the photon count or the R/G ratio (Fig. S10†). Nevertheless, changes in pH were found to have a clear effect on the NV center's charge (Fig. 5a and b), with high pH shifting the R/G distribution towards higher values (from 0.7 to 0.8 R/G ratio; Fig. 5a). Plotting the relation between pH and the mode of the R/G ratio distribution from one field of view (∼100 NDs) showed a roughly linear relationship between pH and R/G ratio for pH values ranging from 4 to 10 (Fig. 5b). Using this relationship, we estimated that we can measure the pH of a solution from the mode of the R/G ratio distribution with an accuracy of ∼0.4 (Fig. 5b).

To test how robust the effect of pH on the R/G ratio is, we considered possible effects of solutions containing chemical species that can strongly interact with the ND surface, such as divalent ions or biological molecules. Specifically, we tested whether pH can still change the R/G ratio in presence of 0.1 M CaCl_2_ or in the presence of a bacterial cell lysate. In both cases, we still found a substantial red shift as we move to higher pH, supporting the potential of the 10 nm ND for pH sensing in complex and biological environments (Fig. S11†).

To explore further the possibility that these NDs could be used as pH sensors, we tested the reversibility of the effect by imaging the same field of view (containing ∼140 NDs) after consecutive immersions of the NDs into acidic and basic solutions. Indeed, the modes of the R/G ratio distribution (Fig. 5c) clearly showed that the effect of pH on the R/G ratio is reversible.

We have also examined whether we have the resolution to detect the increase of the R/G ratio in basic pH on the same ND particle. In Fig. 5d we show the time-traces of the same ND immersed into 2 different solutions (pH 1.2 and pH 12.8). In addition to this change in R/G ratio, we have also observed a decrease in photon count (from 1750 to 1250 photons per 100 ms) at pH 12.8. The correlated increase of R/G ratio and decrease of brightness correspond well with our observation of charge-state conversion on the 50 nm undoped and 40 nm doped NDs (Fig. 2 and 4), with the deviations of the R/G ratio values from 0.6 and 0.9 being attributed to the charge instability in the 10 nm NDs. However, our data indicate that the NV center will stay longer in a charge state (NV^0^ for acidic pH or NV^−^ for basic pH) within 100 ms, leading to changes in the R/G ratio.

The mechanism behind this pH-dependent charge transition of NV is likely to involve deprotonation of ND surface groups (mainly COOH groups generated during ND acid cleaning, and affecting the ND's surface charge), thus creating more negative charges around the NV center.^49^ Our findings confirm previous reports that NDs photophysics is affected by surface chemistry and demonstrate that they can be directly used for pH nanosensing.^32,34^

## Discussion

Using wide-field imaging and automated time-trace analysis, we demonstrate a powerful approach to characterize ensembles of NDs and study their photophysical properties and their potential to act as nanoscale sensors.

By examining large numbers of single NDs in parallel, we were able to detect single NVs in up to 500 NDs per sample, allowing insightful comparisons of the fraction of single emitters in different samples based on their photon count distribution, a task facilitated by the random orientations of the spin-coated NDs on the microscope slide. We also introduce the use of the R/G ratio to analyze the charge state of the NV center, a crucial determinant on the ND photophysical behavior. Our technique can be easily implemented to screen different NDs samples to study the proportion of single NV/ND and the charge stability of the NV center since measuring >100 NDs takes only seconds unlike methods already described.^23,24,50,51^

Since we used a statistical approach to define the photon count provided by a single NV, it is difficult to confirm that a given ND contains only one NV center based on a single observation if no charge transitions are observed. For this reason, photon-correlation experiments are more suited for determining the number of emitting NV centers per particle.^28^ However, this limitation of our approach could be overcome by investigating the orientation of NV center using defocused orientation and position imaging.^52^

Our results on the charge-state instability in 10 nm ND or undoped 50 nm ND confirm previous reports that established the negative impact of ND size and low nitrogen concentration on the stability of the NV^−^.^25,29,30^ Further, our high-throughput approach allowed studies of small but significant subpopulations of the NDs, such as those with dynamic single NV centers; based on 32 dynamic time-traces, we could estimate the lifetimes of the NV^−^ and NV^0^ in NDs. Such a study was not reported before, since confocal or wide-field measurements described lifetimes of NV^−^ from only one single particle.^42,44^

A better investigation of NV charge-state transition in NDs is essential for the development of ND-based biosensing or bio-imaging approaches, as recently reported.^53^ The lifetime measurement methodology we introduced allows automated time-trace analysis from multiple single-NV centres. The closest report to our study of single-NV charge transition is a study by Aslam *et al.* who investigated charge-state transitions of a single NV center in bulk diamond, and measured lifetimes for NV^−^ and NV^0^ (57 ms and 465 ms respectively) three orders of magnitude shorter than what we measured; however, those measurements used substantially different conditions (study in bulk diamond instead of in a ND, and use of a different excitation wavelength: 593 nm), and examined only one NV center.^24^ The presence of a longer component only for NV^0^ in our results might be due to sub-second transitions into the dark NV^+^ state that could slow down the transition from NV^0^ to NV^−^.

We also showed that NDs can be directly used for pH nanosensing. Compared to previous reports on ND pH sensing, our technique is considerably simpler as it does not require ND polymer coating or a microwave generator for spin state manipulation.^27,31,54^ Our demonstration of pH direct reporting at a single-particle level (Fig. 5c) opens the possibility to perform pH nanosensing experiments (such as pH mapping inside living cells) using single-particle tracking. Tracking NDs for pH sensing will be more applicable with the smaller (10 nm *versus* 50–100 nm) and uncoated NDs we used, since the small NDs should result in faster intracellular diffusion, better access to nanoscale structures and lack of any toxicity associated with polymer coatings. Our results also established that NDs can report on pH even in presence of biological molecules; the accuracy (∼0.4) of our measurement is sufficiently high to detect intracellular pH changes such as the transition from lysosomes (pH 5) to cytosol (pH 7).^54^ Our pH sensing experiments also showed a small loss of reversibility during after 5 cycles of pH changes (Fig. 5b); the exact reason for this is unclear, but one possibility is a change in the ND's surface chemistry over time during the pH cycling.

Currently, there is no quantitative model to describe the pH effect on NV's charge state. However, a study by Petráková *et al.* used computational quantum-mechanical modelling predict that a different ND surface termination (oxygen or hydrogen) will change the charge distribution of the surface (*i.e.* different band bending) and, as a result, will change the probability that NV^0^ or NV^−^ states are populated.^14,45^ We speculate that a similar phenomenon may be affecting charge transitions when pH is increased. In basic conditions, functions like carboxylic groups are deprotonated, thus changing the charge distribution on the surface and occupancy probability of NV center charge states. Notably, pH did not significantly impact the R/G ratio in the 44 nm doped NDs (Fig. S9†), consistent with the prediction by Petráková *et al.*, which proposed that only small particles (10–20 nm) could lead to optically detectable transitions of NV's charge because of required proximity to the surface.^32^ Further modelling on oxygen-terminated NDs would be helpful to understand if only band bending is involved, or the phenomenon we report requires more complex models to be properly understood.^55,56^

## Conclusion

In summary, our ability to detect simultaneously hundreds of single NV centers and their photophysics will be extremely valuable for material science and quantum optics applications. Our capability to study dynamic NDs directly and in parallel will enhance our fundamental understanding of NV charge transitions in a nanocrystal and our ability to maintain the negative charge state, which is the state mostly used in ND commercial applications. Our findings should foster more ND applications to single-molecule fluorescence imaging and tracking studies *in vitro* and in living cells, as well as to sensing, *e.g.*, pH monitoring in microfluidics or pH mapping inside biological samples.^57^ Finally, our method should facilitate the development of biosensing assays based on detecting the NV charge state conversion and its dynamics. Such assays will be helped by further study on the effect of bio-functionalization on the charge state, since the NV center can be affected by surface chemistry.

## Conflicts of interest

A. N. K. is a co-founder and shareholder of Oxford Nanoimaging, a company that designs, manufactures and supports single-molecule fluorescence microscopes, including the microscope model used in this study.

## Supplementary Material

NR-012-D0NR05931E-s001
