# Supplementary material for: High-throughput nitrogen-vacancy center imaging for nanodiamond photophysical characterization and pH nanosensing
Source: Nanoscale. 2020 Oct 26;12(42):21821–31. doi: 10.1039/d0nr05931e (PMC8329943; doi:10.1039/d0nr05931e)
Supplement: NR-012-D0NR05931E-s001 [file NR-012-D0NR05931E-s001.pdf]

Electronic Supplementary Material (ESI) for Nanoscale.
This journal is © The Royal Society of Chemistry 2020

Supporting information

High-Throughput Nitrogen-Vacancy Center Imaging for Nanodiamond photophysical characterisation and pH nanosensing

*Maabur Sow, Horst Steuer, Sanmi Adekanye, Laia Ginés, Soumen Mandal, Barak Gilboa, Oliver A. Williams, Jason M. Smith, Achillefs N. Kapanidis**

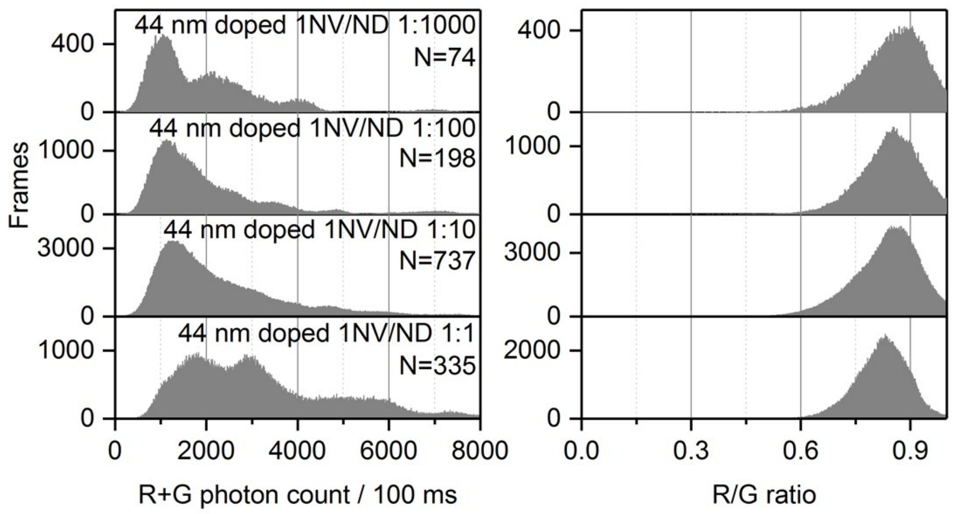

Fig. S1: distribution of photon count and R/G ratio in different NDs dilutions. We previously checked that the NDs solutions are not aggregated by dynamic-light scattering or single-particle tracking analysis. However, there is a possibility that aggregates may have formed when NDs were deposited on the slide, which could make the first mode of the photon count distribution (i.e. 1000 photons/100 ms from Fig. 3a) originating from two or more NV centers instead of a single NV. To test this hypothesis, we measured the distribution of the photon count from different dilutions of the 44-nm 1NV/ND ND solution. If the mode at 1000 photons/100 ms is due to aggregates, the highest dilution (1:1000) should show a mode inferior to 1000 photons/100 ms. Results in the Fig. above shows that the most diluted sample (1:1000, see top row) still has a clear peak at 1000 photon/100 ms while the undiluted sample (1:1) displays a first maxima at 2000 photon/100 ms (see bottom row). The fact that this mode at 1000 photons/100 ms becomes even more distinct at a low dilution is consistent with the data from the 10 and 50-nm NDs, which support our previous deduction that the photon count of 1000 photons/100 ms corresponds to the emission of single NV- center. N is the number of fluorescent NDs observed and “Frames” is the frequency of the measurement from a ND (see Fig. 1 for details). The 44-nm doped NDs (1NV/ND) were diluted in deionized water and imaged in air with 532-nm excitation 7.8 kW cm-2 with 100 ms exposure for 25 s.

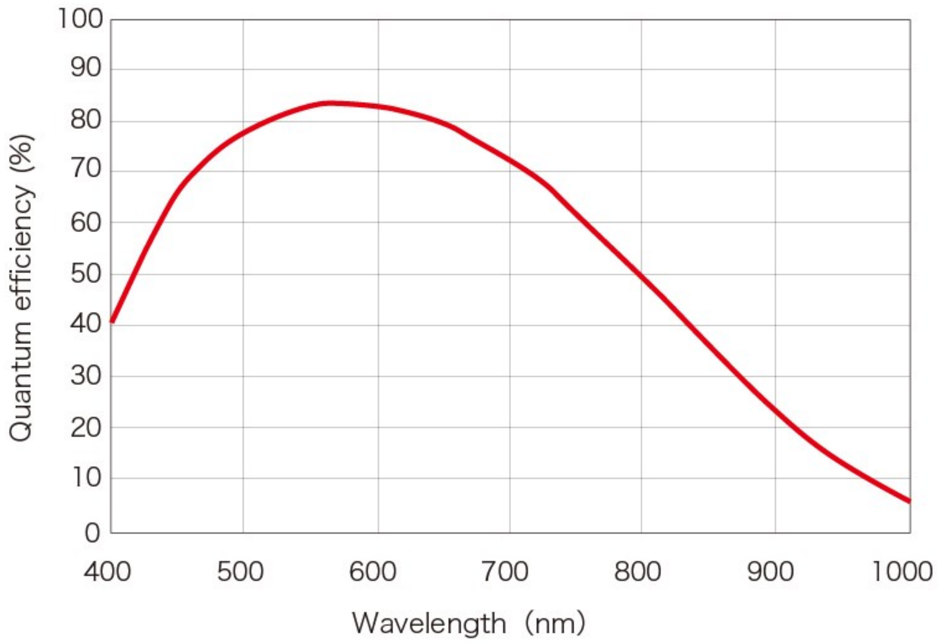

Fig. S2: quantum efficiency of the scientific CMOS camera implemented in the wide-field microscope. The quantum efficiency of the sCMOS is at its maximum (>80%) between 520 and 650 nm which corresponds to the main emission band of NV^0. Such a difference in quantum efficiency could explain why we measured a higher total photon count (green + red channel) of NV^0 50 % superior to NV^-. Data obtained from the website of the sCMOS' manufacturer on 1-jan-19 (https://www.hamamatsu.com/jp/en/C13440-20CU.html#1328482115119).

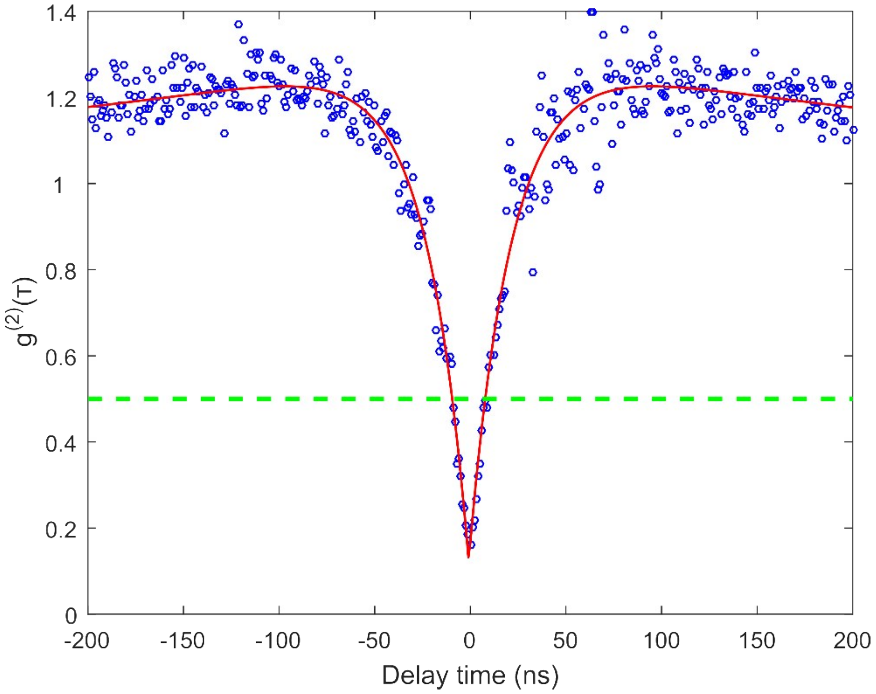

Fig. S3: second order correlation function from single NV center in a 44-nm doped NDs (1NV/ND). The presence of single NV center was also confirmed by photon correlation experiments. The drop of the second order correlation function (*i.e.* antibunching) below 0.5 confirms that the emitter observed is a single photon source. Antibunching confirming the presence of single NV center was observed in 23 % of the NDs measured (N=311, see example above).

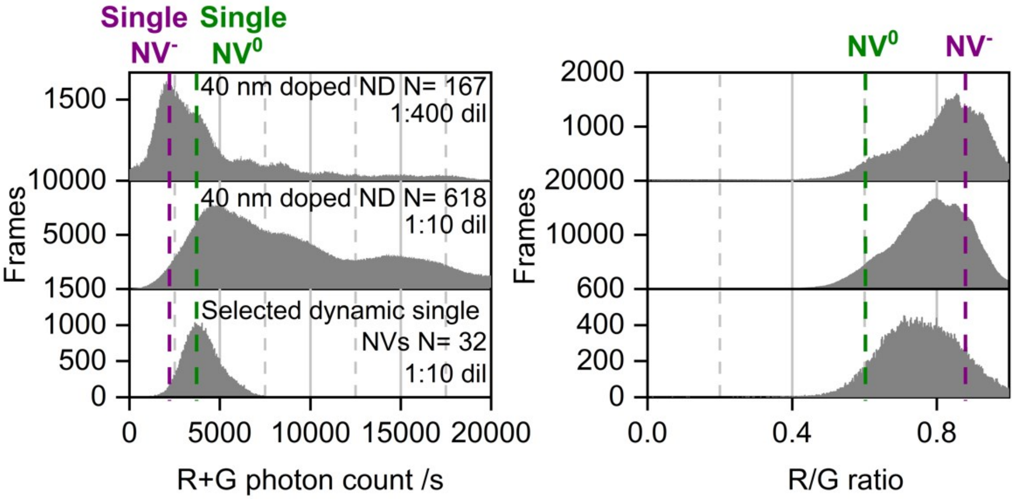

Fig. S4: distribution of photon count and R/G ratio in different NDs samples. For the study of dynamic NDs, the nanoparticles were imaged with 1 s exposure time and a reduced laser excitation (3.4 kW cm^{-2}) for 20 to 60 min. The photon count distribution of the diluted 40-nm doped NDs (1:400 see top row) allowed us to identify the level from single NV^- and NV^0 as described previously in the first part of the result section (see Fig. 2 and 3). To increase the probability to obtain time-traces of dynamic single NV centers per field of view, we increased the ND concentration (see middle row, dilution 1:10). Time-traces corresponding to single NV centers (dynamic and stable ones) were manually selected based as described in materials and methods. The selected dynamic single NVs (see bottom row) contains mainly time-averaged NV^-/NV^0 and NV^0 as shown by the R/G ratio distributions. The reason why most of stable NV^- traces were excluded is that the intensity of NV^- in the green channel was approaching zero, which was causing error in the HMM analysis. Nevertheless, the dynamic ones provided enough dwells in the NV^- state for the HMM algorithm to detect and populate this state as shown in Fig. 4c. N is the number of fluorescent NDs observed and "Frames" is the frequency of the measurement from a ND (see Fig. 1 for details).

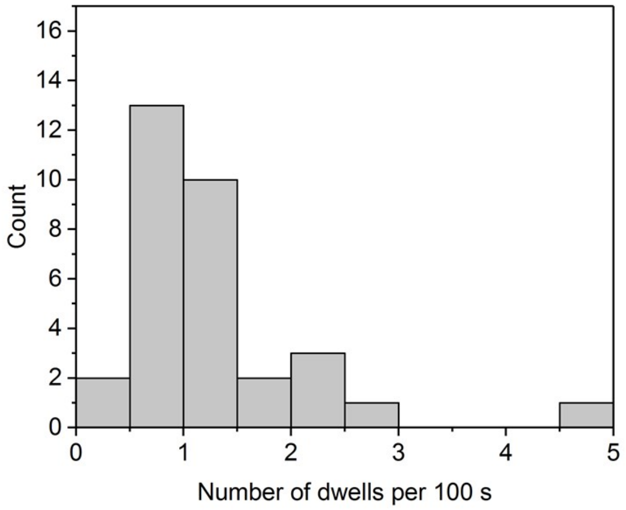

Fig. S5: distribution of the number of dwells 100/s from the dynamic time-traces.

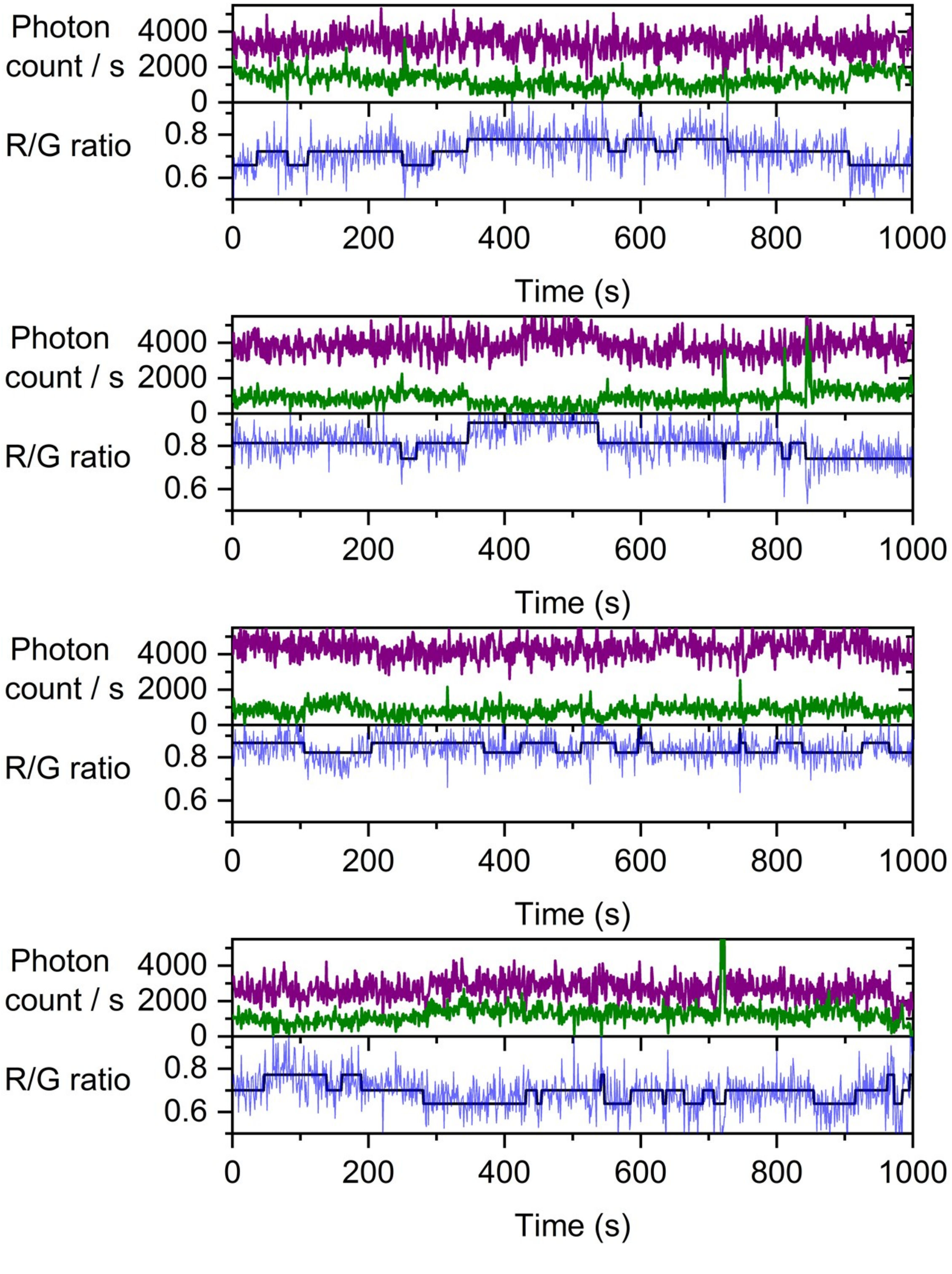

Fig. S6: Typical moderate-fluctuation dynamic traces. The immobilized NDs were imaged in air using 532-nm excitation 3.4 kW cm^{-2} and 1 s exposure for 20 min to 60 min.

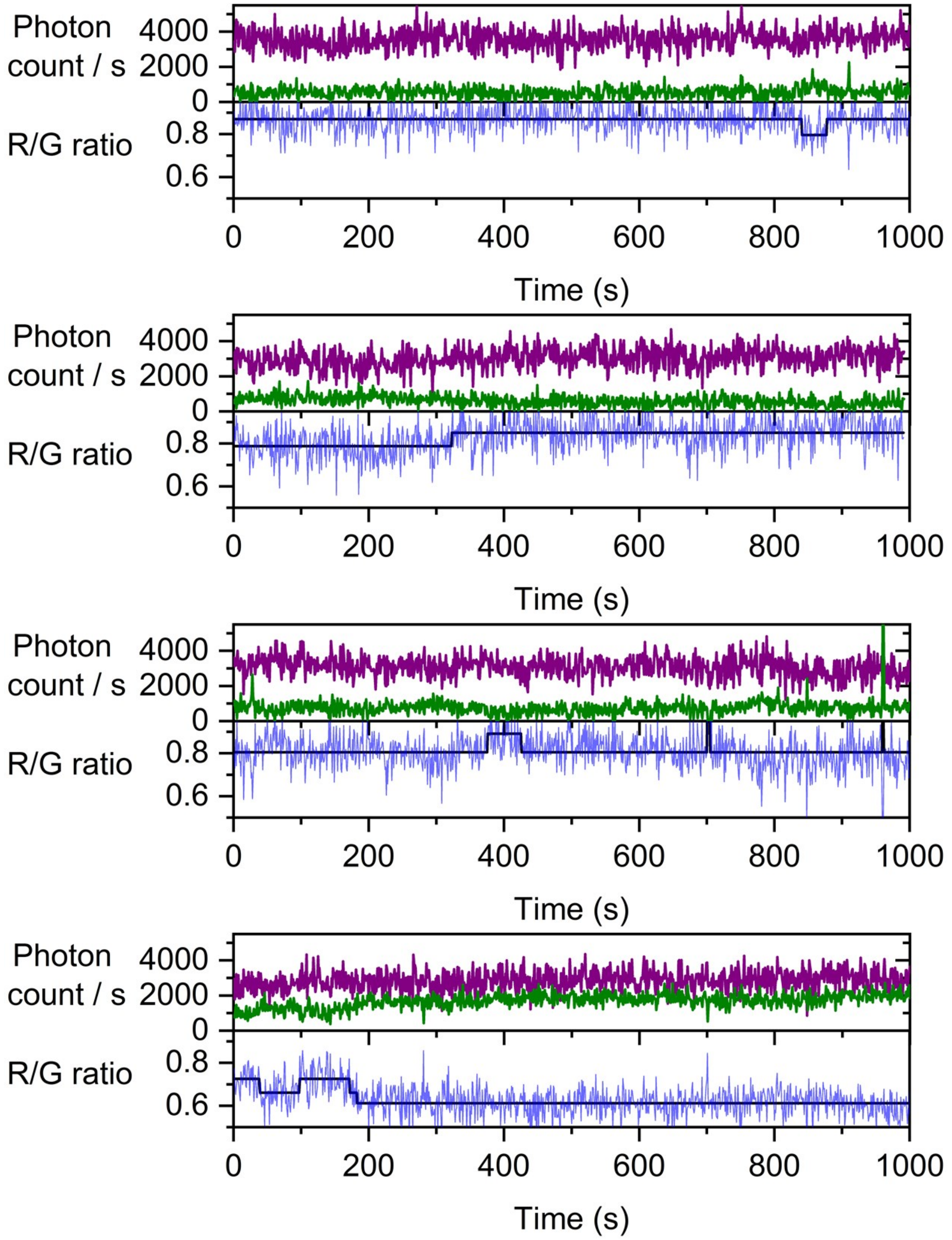

Fig. S7: Typical rare-fluctuation dynamic traces. The immobilized NDs were imaged in air using 532-nm excitation 3.4 kW cm^{-2} and 1 s exposure for 20 min to 60 min.

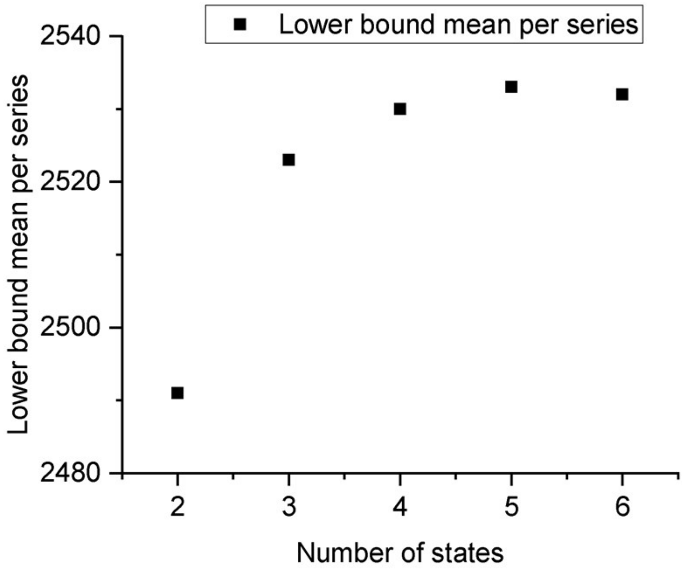

Fig. S8: plot of the mean values for lower bound per series for the models with different number of states as extracted from the HMM analysis. As described by van de Meent *et al.*[47], the lower bound is used to compare the fitting of each model (*i.e.* one to 6 states). The higher the lower bound is and the better the fit is. As presented above, the 3-state model shows the largest increase in the lower bound (+30). Additional states led to only smaller improvements, for this reason and because we mainly observed three states in our analysis of static traces, we decided to use a 3-state model. The standard deviation is 1110 per point.

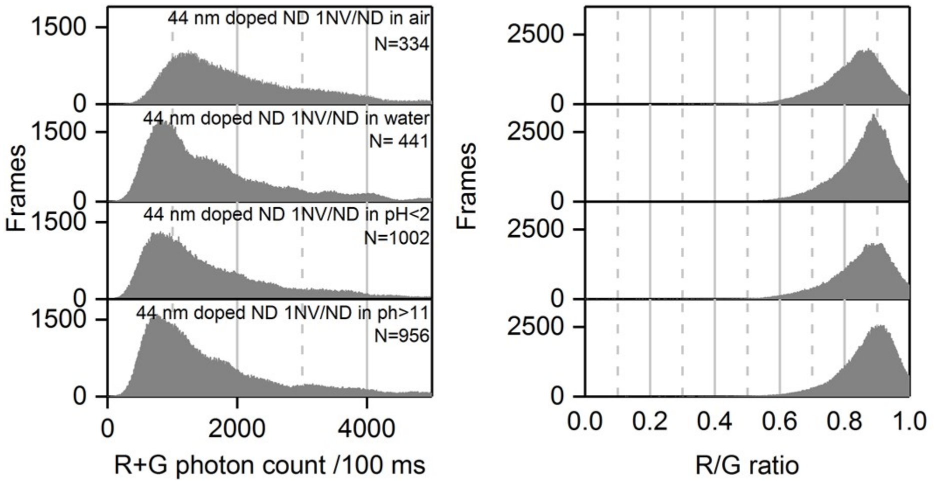

Fig. S9: distribution of photon count and R/G ratio from 44-nm doped NDs (1NV/ND) in different solutions. The effect of pH in changing the charge state could not be reproduced in the 44-nm NDs. As shown in the Fig. above, the two bottom rows (in acid and base) show no significant change in the photon count distribution and R/G ratio as observed in the 10-nm NDs (see Fig. 5). A 0.01 M HCl solution was used for pH <2 measurement and a 0.1 M NaOH for pH >11. N is the number of fluorescent NDs observed and “Frames” is the frequency of the measurement from a ND (see Fig. 1 for details). The immobilized NDs were imaged with 532-nm excitation 7.8 kW cm^{-2} 100 ms exposure for 25 s.

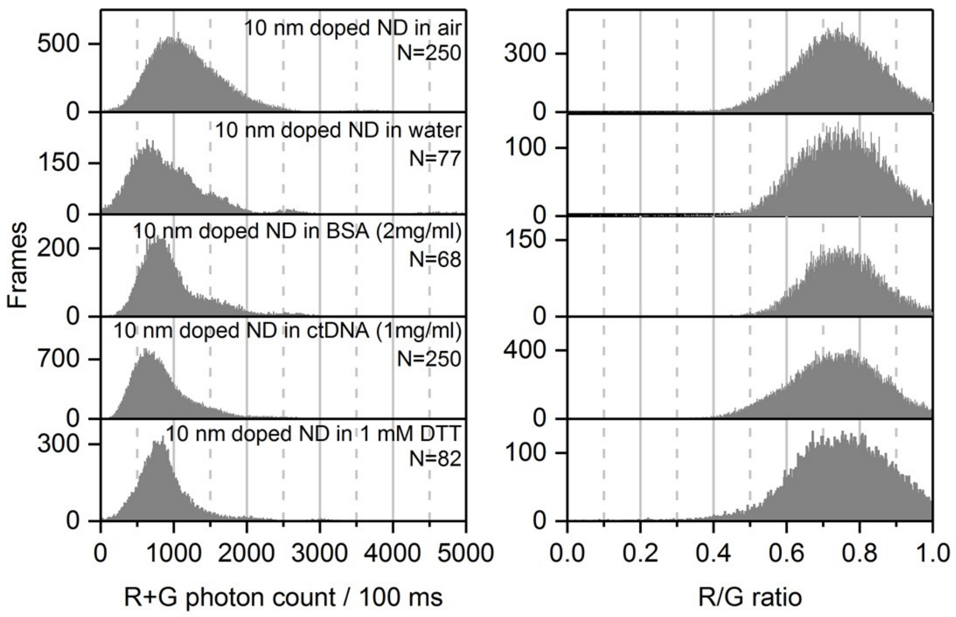

Fig. S10: distribution of photon count and R/G ratio from 10-nm doped NDs in different solutions. No effect of different solutions (reducing agent DTT: Dithiothreitol, BSA: bovine serum albumin, and ctDNA: calf thymus DNA) on the charge state was found on the 10-nm NDs. As shown in the Fig. above, the three bottom rows (in DTT, BSA and DNA) show similar photon count distribution and R/G ratio compared to water (second row). N is the number of fluorescent NDs observed and “Frames” is the frequency of the measurement from a ND (see Fig. 1 for details). The immobilized NDs were imaged with 532-nm excitation 7.8 kW cm^{-2} and 100 ms exposure for 25 s.

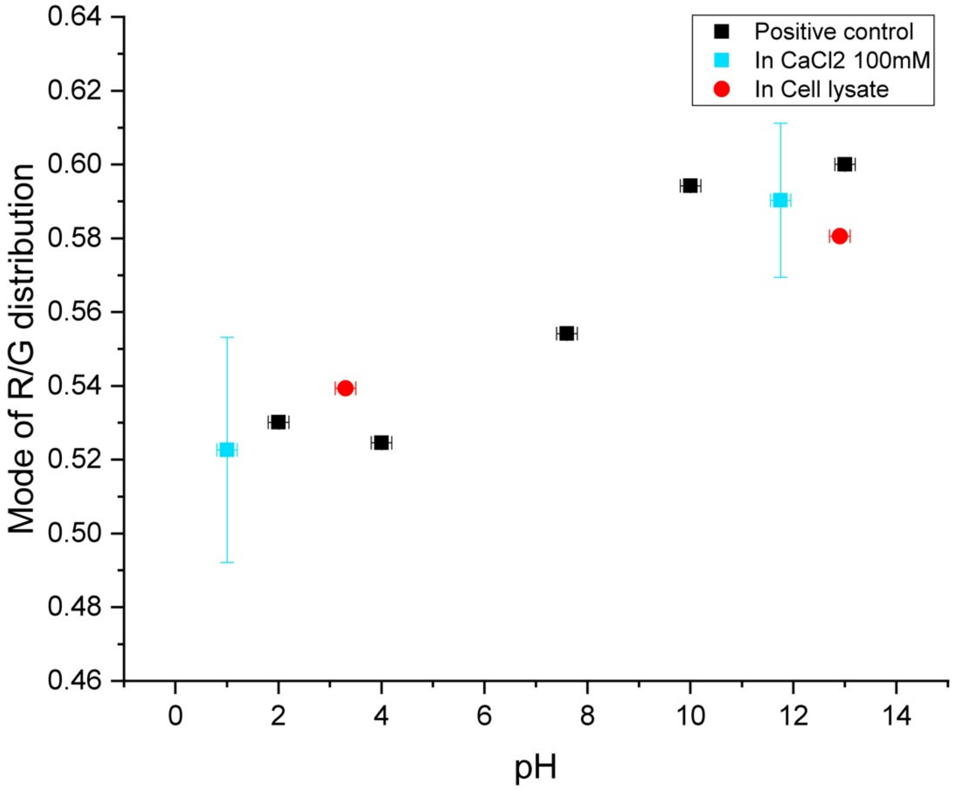

Fig. S11: Addition of cell lysates or $CaCl_2$ to NDs does not affect significantly the main pH effect on the R/G ratio. The values of the mode of the R/G distribution for pH 1-4 and 11-13 in cell lysate (from *E. coli*) and in 0.1 M $CaCl_2$ show no significant difference (+/- 0.02) compared to the positive control containing only buffer solutions. The R/G ratio values for the positive control are different from the one plotted in Fig. 5b because a different microscope was used for all the measurement presented in the Fig. above. All measurements are from more than 150 NDs recorded in one field of view, while an average of duplicate (two fields of view) is shown for $CaCl_2$. X error bars are from the pH meter's accuracy; Y error bars are the accuracy from the Gaussian fit except for $CaCl_2$, which represent the difference between the duplicates.The larger error bar for $CaCl_2$ is due to a significant temperature difference inside the chamber between the two recordings (+/- 5°C).

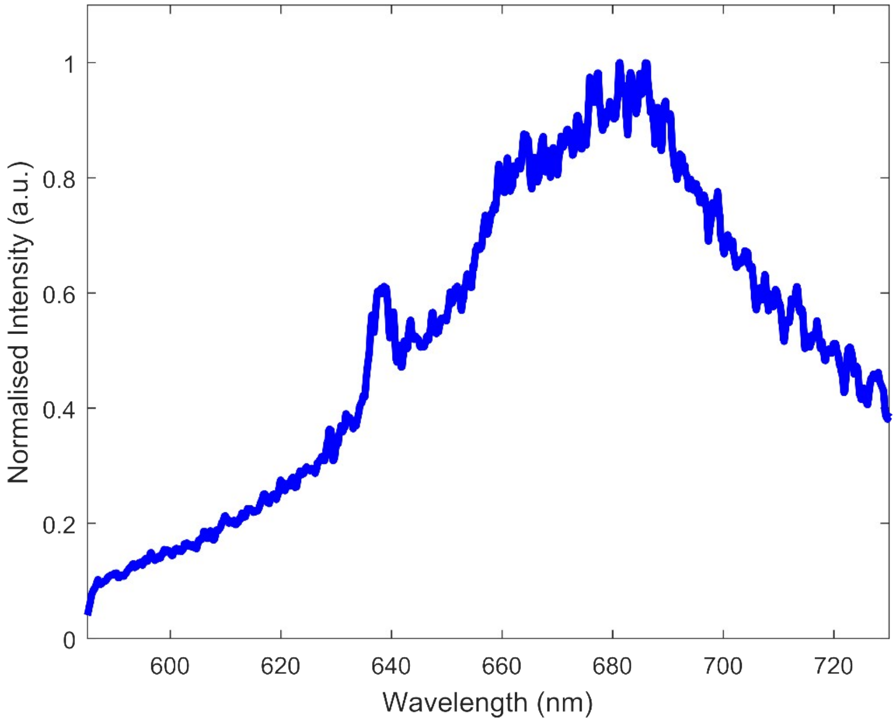

Fig. S12: emission spectrum of NV^- from the 44-nm doped NDs (1NV/ND) with 532-nm laser excitation. The presence of NV center in NDs is verified by the manufacturer in the doped samples (10, 40, 44 and 200-nm). The emission spectra of four 44-nm doped NDs were also measured using a confocal set-up similar to the one described in reference [41]. As shown above, they all show typical NV^- emission spectra with the characteristic zero-phonon-line at 637 nm.
